# Zonisamide Ameliorated the Apoptosis and Inflammation in Cerebellar Tissue of Induced Alcohol Addiction Animal Model

**DOI:** 10.3390/life14070795

**Published:** 2024-06-24

**Authors:** Fırat Aşır, Fikri Erdemci, Zuhal Çankırı, Tuğcan Korak, Süreyya Özdemir Başaran, Özge Kaplan, Özkan Yükselmiş, Nilüfer Dönmezdil, Hayat Ayaz, Şehmus Kaplan, Selçuk Tunik

**Affiliations:** 1 Department of Histology and Embryology, Medical Faculty, Dicle University, 21280 Diyarbakır, Turkey; 2 Department of Medical Biology, Medical Faculty, Kocaeli University, 41001 Kocaeli, Turkey; 3 Department of Andrology, Gazi Yasargil Education and Research Hospital, Health Sciences University, 21090 Diyarbakir, Turkey; 4 Division of Physical Medicine and Rehabilitation, Diyarbakır Dağ Kapı State Hospital, 21100 Diyarbakır, Turkey; 5 Department of Audiology, Faculty of Health Sciences, Mardin Artuklu University, 47200 Mardin, Turkey

**Keywords:** zonisamide, cerebellum, neuroinflammation, inflammation, apoptosis

## Abstract

This study investigated the effects of zonisamide treatment on cerebellar tissues in an experimental alcohol addiction (AA) model and its potential mechanisms of action, particularly regarding apoptotic protease activating factor-1 (APAF-1) and tumor necrosis factor-alpha (TNF-α) expression. Thirty rats were divided into three groups: sham, ethanol (EtOH), and EtOH + zonisamide. AA was induced by administering 6 cc of EtOH orally every 8 h for 4 days. Zonisamide (100 mg/kg) was given to rats once daily before EtOH administration. Motor defects were evaluated using an open field maze. Serum TNF-α levels were measured from blood samples. Cerebellar sections were processed for histological examination and immunostained for APAF-1 and TNF-α. Protein interaction networks were constructed using Cytoscape, and functional annotations were performed with ShinyGO (version 0.80) software. The traveled area in the EtOH group was significantly reduced compared to the sham group (*p* = 0.0005). Rats in the EtOH + zonisamide group covered a larger area, with zonisamide treatment significantly improving locomotor ability compared to the EtOH group (*p* = 0.0463). Serum TNF-α levels were significantly elevated in the EtOH group compared to the sham group (*p* < 0.0001) and were significantly decreased in the EtOH + zonisamide group compared to the EtOH group (*p* = 0.0309). Regular cerebellar histological layers were observed in the sham group, while EtOH induction caused loss of cerebellar tissue integrity, neuronal degeneration, vascular dilatation and congestion, reduced myelin density, and neuropils in the EtOH group. Zonisamide treatment improved these pathologies, enhancing myelination and neuropil formation. Negative APAF-1 and TNF-α expressions were observed across cerebellar layers in the sham group. Due to EtOH toxicity, APAF-1 and TNF-α expression were upregulated in the EtOH group compared to the sham group (*p* < 0.001 for both). Zonisamide treatment downregulated these protein expressions in the EtOH + zonisamide group compared to the EtOH group (*p* < 0.001 and *p* = 0.0087, respectively). APAF-1 was primarily associated with AA through antifolate resistance, endopeptidases, and the interleukin-1 pathway, while TNF-α was predominantly enriched in infections and choline-binding, indicating zonisamide’s impact on immune and inflammatory pathways. In conclusion, zonisamide treatment significantly mitigated ethanol-induced cerebellar damage and inflammation in an AA model. Zonisamide improved locomotor function and reduced serum TNF-α levels, as well as APAF-1 and TNF-α expression in cerebellar tissues. These findings suggest that zonisamide exerts its protective effects by modulating immune and inflammatory pathways, thereby preserving cerebellar integrity and function.

## 1. Introduction

The cerebellum, an integral component of the central nervous system, is pivotal for motor coordination and balance [1]. Its intricate somatotopic organization establishes direct connectivity with the primary motor and sensory cortices, thereby orchestrating a sophisticated network for sensorimotor integration. In addition to its fundamental role in motor and sensory processing, the cerebellum is actively involved in various perceptual tasks, including the interpretation of visual, auditory, tactile, and nociceptive stimuli. Furthermore, the cerebellum plays a critical role in cognitive and emotional functions, forming connections with the prefrontal cortex and limbic system, and thus significantly contributing to numerous mental and emotional processes [2,3]. Heavy alcohol intake exerts deleterious effects on the cerebellum, leading to cellular apoptosis, tissue atrophy, and cognitive dysfunction [4]. Alcohol addiction (AA) is a significant health risk, contributing notably to liver cirrhosis and ranking as the third leading cause of premature mortality in European countries. Moreover, AA is implicated in 60 different illnesses and pathological conditions, with approximately 2.3 million deaths annually attributed to alcohol consumption [5]. The binge ethanol model for studying alcohol dependence has been extensively explored in preclinical research. Faingold et al. [6] determined that administering ethanol every 8 h over a four-day period effectively induces maximal signs of ethanol intoxication. Nixon et al. also found that administering ethanol over four days induces alcohol addiction in rats. In a study by Crews et al. [7], rats received intragastric ethanol three times daily for four days, resulting in blood alcohol levels ranging from 250 to 400 mg, causing significant intoxication.

Numerous studies have shown that binge ethanol consumption leads to brain damage [7,8]. This damage begins in the frontal olfactory bulb after two days and progressively affects additional brain regions over time. Research consistently demonstrates that peak brain damage occurs shortly after the final dose on the fourth day [9]. Although the exact mechanism of AA is unclear, several factors contribute to its etiology. AA causes alcohol toxicity in organs, leading to cellular tissue damage and death [5]. Researchers have shown a relationship between proinflammatory cytokines and alcohol addiction. An experimental study revealed that long-term alcohol consumption exacerbates the inflammation process and elevates TNF-α (tumor necrosis factor-alpha) levels in rats’ serum, leading to tissue damage [10]. A clinical study found an association between alcohol consumption and serum TNF-α levels in 30 male alcohol-addicted patients. The authors suggested that a high level of TNF-α indicates the longevity and severity of alcohol addiction [11]. Additionally, alcohol consumption plays an important role in the activation of mitochondrial-dependent apoptosis through APAF-1 (apoptotic protease activating factor-1) [12,13]. Wang et al. [14] showed that high-dose alcohol consumption led to the production of reactive oxygen species (ROS), eventually causing apoptosis in mouse primary cardiomyocytes. Similarly, Hajnóczky et al. [15] found that prolonged alcohol consumption disrupted the membrane permeabilization of mitochondria, allowing for the induction of apoptosis in cardiac cells.

Anticonvulsants, initially developed for managing epilepsy, have emerged as a promising avenue in the treatment of alcohol addiction and dependency [16]. Recent investigations underscore the efficacy of antiepileptic agents in mitigating alcohol intake, as evidenced by clinical trials [17,18]. Zonisamide, an anticonvulsant sharing mechanistic similarities with topiramate [19], has been proposed as a potential therapeutic intervention for AA. Its mechanism of action involves the reinforcement of gamma-aminobutyric acid (GABA)-mediated neurotransmission. GABA, a neurotransmitter intricately involved in the regulation of anxiety, sleep, and muscle tone [20], plays a pivotal role in attenuating alcohol cravings and the concomitant anxiety associated with alcohol withdrawal. Arias et al. studied the role of zonisamide in alcohol dependence and found that it could be used to treat alcohol dependence [21]. A clinical study indicated that zonisamide reduced the stimulatory impacts of AA in patients [22]. Previous studies have used various dosages of zonisamide across different species (rabbits at 3–30 mg/kg [23,24], mice at 0, 25, 50 mg/kg [25], and rats at 25–50 mg/kg [25,26]), indicating its effectiveness against neurological complications. Kumar et al. [27] studied a higher dose of zonisamide (100 mg/kg) in rats and demonstrated significant neuroprotective potential against seizures by mitigating oxidative stress, inflammation, and neuronal death, indicating its promise as a neuroprotective agent for epilepsy and other neurodegenerative diseases.

Although the impacts of zonisamide on AA have been previously investigated, there is no study on the effects of zonisamide on cerebellar pathology in induced AA animal models. This study aimed to elucidate the impact of zonisamide in mitigating cerebellar cellular inflammation and apoptosis subsequent to AA by assessing the expression levels of APAF-1 and TNF-α via immunostaining and bioinformatic methods.

## 2. Materials and Methods

### 2.1. Animal Housing and Experimental Design

Thirty male Wistar albino rats were allowed access to water and food ad libitum and housed in cages (12/12 dark/light period, 23 ± 1 °C). Zonisamide (100 mg in each capsule) was purchased from Gerenica^®^ company. Generica (Genveon Drug Company, Sarıyer, Istanbul, Turkey). The experimental design was modified according to the study by Faingold et al. [6]. Thirty rats were categorized into three groups (ten rats per group):

1. Sham group: 6 cc of physiological saline was given orally to rats 3 times a day for 4 days at 8 h intervals.
2. Ethanol (EtOH) group: 6 cc of EtOH was administered orally to rats 3 times a day for 4 days at 8 h intervals.
3. EtOH + zonisamide group: Rats were given 6 cc of EtOH orally 3 times a day for 4 days at 8 h intervals. One hour before each EtOH administration, 100 mg/kg of zonisamide was administered to the rats once a day for 4 days.

### 2.2. Open Field Maze

This test was conducted according to the procedures previously described by Seibenhener et al. [28]. A white, high-density, non-porous plastic chamber with dimensions of 50 × 50 × 38 cm was used, equipped with a video camera positioned above it. The rats were initially placed in the center of the chamber, and their movements were recorded using tracking software (EthoVision XT (version 17.5, Noldus Inc., Leesburg, VA, USA)). The rats were allowed to move freely throughout the maze for 10 min while the software tracked their movements. The total ambulatory distance (cm) was evaluated to measure the locomotor ability of the rats. 

### 2.3. Measurement of Serum TNF-α

All animals were euthanized with an intramuscular injection of 90 mg/kg ketamine (Ketasol; Richter Pharma AG, Feldgasse 19, 4600 Wels, Austria) and 8 mg/kg xylazine (Rompun; Bayer, Germany) after the maze test. Intracardiac blood samples were collected from rats and analyzed for serum TNF-α content via ELISA kit (catalog no: E-EL-R2856, Elabscience Bionovation Inc., Houston, TX, 77079, USA, reactivity: rat). Blood samples from each rat were centrifuged at 2000 rpm for 10 min, and the supernatant was collected. Serum TNF-α (pg/mL) content was measured according to the manufacturer’s instructions.

### 2.4. Tissue Preparation

Cerebellar tissues were removed for histological examination. The dissected samples were further placed in zinc formalin, dehydrated using a series of alcohol solutions, and then embedded in paraffin wax. In total, 5 µm sections were stained with hematoxylin eosin and Luxol fast blue dye.

### 2.5. Immunohistochemical Examination

Cerebellar sections were subjected to dewaxing, hydration in graded alcohol solutions, and rinsing with distilled water. To inhibit endogenous peroxidase activity, 3% hydrogen peroxide (H_2_O_2_) was applied to the slides. Following washing in phosphate-buffered saline (PBS), the sections were incubated overnight at a temperature of 4 °C with primary antibodies APAF-1 (catalog no: sc-65891, Santa Cruz Biotechnology, Inc. 10410 Finnell Street Dallas, TX 75220, USA, 1/100) and TNF-α (catalog no: sc-133192, Santa Cruz Biotechnology, USA, 1/100). Sections were biotinylated and reacted with peroxidase solution for 15 min. After PBS rinsing, diaminobenzidine (DAB) chromogen was used to observe color changes. The reactions were stopped using a PBS solution, and the sections were counterstained with hematoxylin dye. Subsequently, the slides were mounted and imaged using a Zeiss Imager A2 light microscope [29].

### 2.6. Semi-Quantitative Histological Scoring

All images underwent processing and quantification using the ImageJ software (version 1.53, http://imagej.nih.gov/ij (accessed on 13 April 2024). The APAF-1 and TNF-α staining intensity was measured by following the method described by Crowe et al. [30]. The quantitative analysis involved examining ten fields from each specimen within the respective groups [31]. In specimens, a brown color indicated positive expression of the antibody of interest, while a blue color signified negative expression. The signal intensity (expression) in a field was determined by dividing the intensity of the antibody of interest by the entire specimen area. The staining area/whole area ratio was computed for each specimen across ten fields. An average value was calculated for groups and subjected to semi-quantitative immunohistochemistry scoring.

### 2.7. Functional Enrichment Analysis

Functional enrichment analyses were conducted to elucidate the mechanisms of action of zonisamide in AA through APAF-1 and TNF-α proteins. Initially, the protein−protein interaction (PPI) networks of target proteins were intersected with the AA-PPI network through Cytoscape software v3.10.1 using STRING data (maximum additional interactors: 100 and cut-off: 0.4). The numbers of shared proteins were visualized using the Venny 2.1 tool [32]. Subsequently, the Kyoto Encyclopedia of Genes and Genomes (KEGGs) pathway enrichment and gene ontology (GO) function annotation analyses were performed using common proteins in the PPI network through ShinyGO 0.80 [33]. Pathways exhibiting a false discovery rate (FDR) below 0.05 were categorized using fold enrichment. Consequently, KEGGs and GO molecular function (MF) box plots were generated.

### 2.8. Statistical Analysis

Statistical analysis was performed using the IBM SPSS 25.0 software (IBM, Armonk, New York, NY, USA) (All graphs were constructed via IBM software.). Data distribution was assessed using the Shapiro–Wilk test and recorded as median (interquartile range—IQR) since data were not distributed normally. The non-parametric Kruskal−Wallis test compared more than two groups, with the post hoc Dunn test applied due to the limited number of animals in each group. Statistical significance was determined for values with *p* < 0.05.

## 3. Results

### 3.1. Zonisamide Elevated Locomotor Activities of Rats

Rats were tested for locomotor abilities in an open field maze. The total ambulatory distance of the rats in the maze is shown in Table 1. The rats in the EtOH group covered significantly less distance compared to the sham group (*p* = 0.0005), indicating a clear negative impact of ethanol on locomotor activity. After zonisamide treatment, rats traveled significantly more distance compared to the EtOH group (*p* = 0.0463). These results suggest that zonisamide may mitigate the locomotor impairments induced by ethanol exposure, highlighting its potential therapeutic benefit.

### 3.2. Zonisamide Decreased the Serum TNF-α Levels

**Figure 1:**
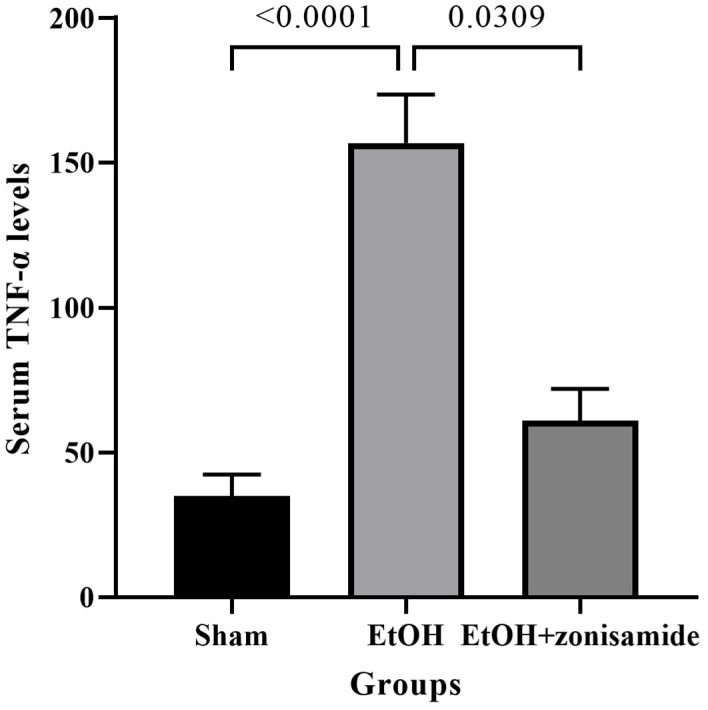
Serum TNF-α levels in blood samples of sham, EtOH, and EtOH + zonisamide groups. EtOH: ethanol.

Serum TNF-α levels were significantly increased in the EtOH group compared to the sham group (*p* < 0.0001). After zonisamide treatment, serum TNF-α levels were significantly decreased in the EtOH + zonisamide group compared to the EtOH group (*p* = 0.0309), suggesting that zonisamide effectively alleviated ethanol-induced inflammation (Figure 1).

### 3.3. Zonisamide Improved Histopathology of Cerebellar Tissue

**Figure 2:**
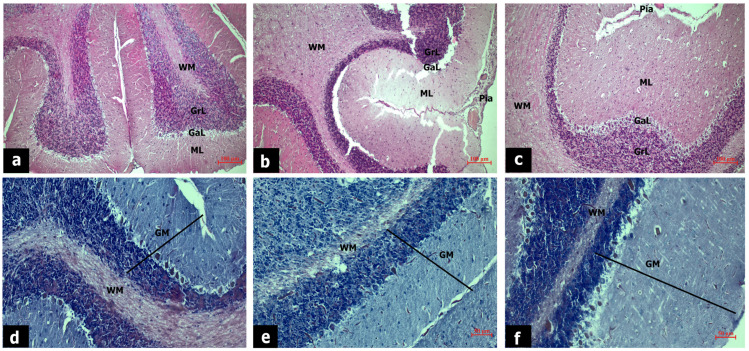
Hematoxylin eosin staining of cerebellar tissues of sham (**a**), EtOH (**b**), EtOH + zonisamide (**c**) group, scale bar: 100 µm, magnification: 10×. Luxol fast blue staining of cerebellar tissues of sham (**d**), EtOH (**e**), EtOH + zonisamide (**f**) group, scale bar: 50 µm, magnification: 20×. GM: gray matter, WM: white matter, ML: molecular layer, GaL: ganglionic layer, GrL: granular layer, Pia: piamater.

Histochemical staining of cerebellar tissue sections is shown in Figure 2. In the cerebellar sections of the sham group, neurons in the molecular, ganglionic, and granular layers appeared normal (Figure 2a). In the EtOH group, vascular dilatation and congestion in the meninges, loss of tissue integrity, neuronal degeneration in the molecular layer, and apoptosis in Purkinje cells were observed (Figure 2b). In the zonisamide-treated group, vascular congestion in the meninges persisted, but pathologies in the molecular and ganglionic layers improved (Figure 2c).

In cerebellar sections stained with Luxol fast blue dye, densely myelinated axons in the gray and white matter were stained blue. Neuropils in the white matter were intensely stained with eosin in the sham group (Figure 2d). In the EtOH group, myelin density in the gray matter and neuropils in the white matter decreased compared to the sham group (Figure 2e). Zonisamide treatment promoted myelination in the white and gray matter and enhanced neuropil formation in the white matter (Figure 2f).

### 3.4. Zonisamide Prevented Apoptosis and Neuroinflammation in Cerebellum

**Figure 3:**
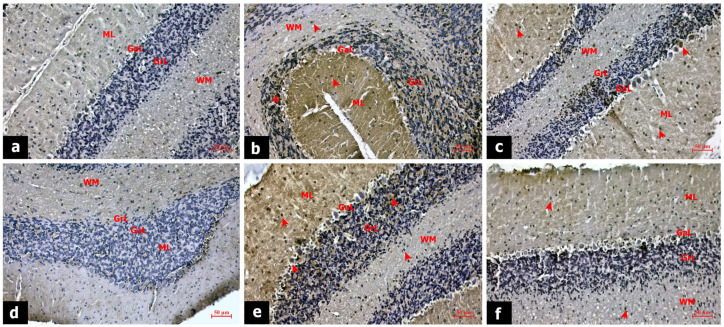
APAF-1 immune staining of cerebellar tissues of sham (**a**), EtOH (**b**), EtOH + zonisamide (**c**) group. TNF-α staining of cerebellar tissues of sham (**d**), EtOH (**e**), EtOH + zonisamide (**f**) group. Scale bar: 50 µm, magnification: 20×. Arrowhead: positive expression, GM: gray matter, WM: white matter, ML: molecular layer, GaL: ganglionic layer, GrL: granular layer, Pia: piamater.

Immunostaining of cerebellar tissue sections is shown in Figure 3. The effects of AA on cell survival were monitored by APAF-1 expression. In the sham group, APAF-1 expression was generally negative in the molecular, ganglionic, and granular layers of the cerebellar cortex and white matter (Figure 3a). Alcohol toxicity induced cell death and increased APAF-1 expression in the EtOH group. The APAF-1 expression was intense in the basket and star cells of the molecular layer, Purkinje cells, and granular cells. The APAF-1 expression was also increased in neuroglia cells in the white matter (Figure 3b). In the EtOH + zonisamide group, APAF-1 expression was decreased in the cerebellar cortex and white matter, suggesting that zonisamide treatment promoted cell survival in the cortical layers (Figure 3c). The neuroinflammatory effect of AA was analyzed by TNF-α expression. In the sham group, TNF-α expression was negative (Figure 3d). In the EtOH group, TNF-α expression increased due to inflammation, especially in neurons in the molecular layer and Purkinje cells (Figure 3e). After zonisamide treatment, TNF-α expression in the EtOH + zonisamide group became mostly negative in ganglionic and granular layers and neuroglia in the white matter (Figure 3f).

### 3.5. Zonisamide Downregulated Expression of APAF-1 and TNF-α

**Figure 4:**
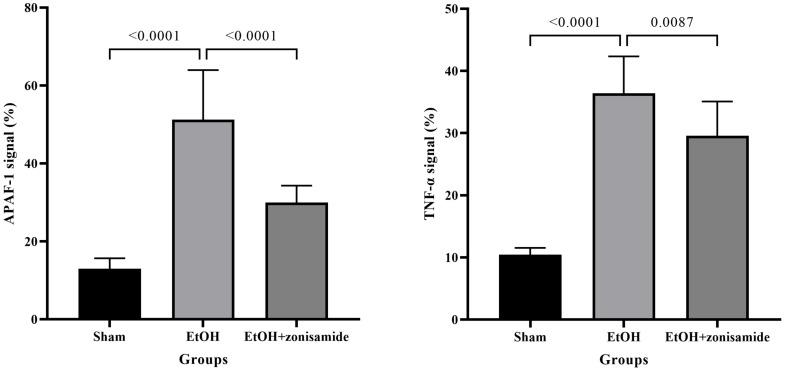
Semi-quantitative scoring of APAF-1 (**left**) and TNF-α (**right**) immune staining. Zonisamide treatment significantly decreased the expression of proteins after alcohol toxicity.

A semi-quantitative analysis of APAF-1 and TNF-α expressions is shown in Figure 4. AA induced the upregulation of APAF-1 and TNF-α expression in the cerebellar cortex; however, zonisamide treatment significantly lowered their expression in cerebellar tissues.

### 3.6. AA Is Molecularly Associated with APAF-1 and TNF-α Pathways

**Figure 5:**
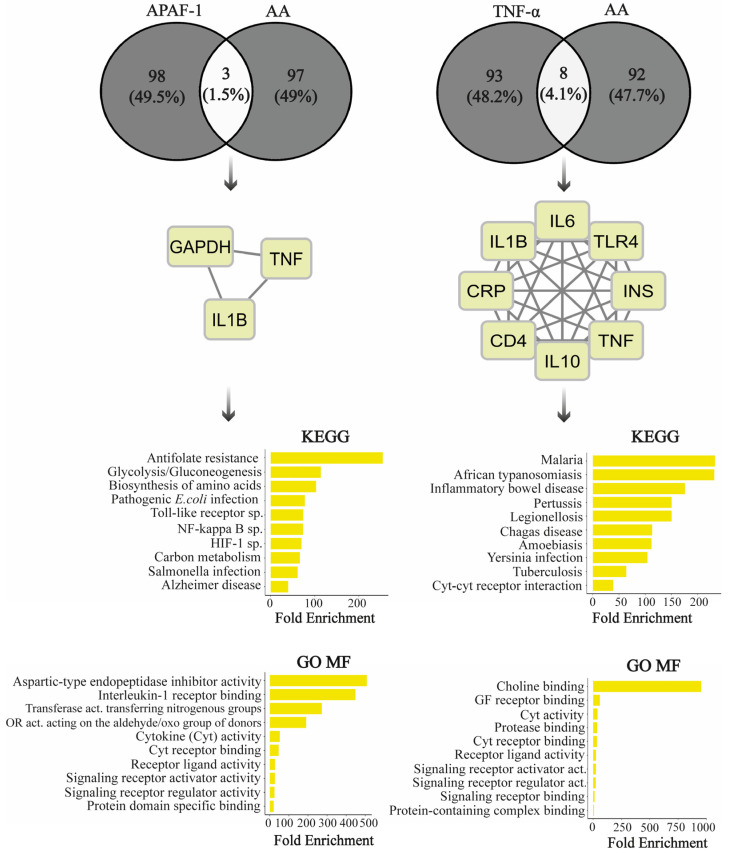
KEGGs pathway and GO molecular function (MF) analysis based on AD correlated interactors of Apaf-1 and TNF-α. The Venn diagram represents the quantities of shared and non-shared proteins in the PPI networks. Functional annotations of shared PPI networks were illustrated using box plots, where a larger box indicates stronger enrichments (FDR < 0.05). CD4: cluster of differentiation 4, CRP: C-reactive protein, Cyt: cytokine, GAPDH: glyceraldehyde 3-phosphate dehydrogenase, IL: interleukin, INS: insulin, OR: oxidoreductase, Sp: signaling pathway, TLR4: toll-like receptor 4, TNF: tumor necrosis factor.

The results of the analyses revealed the pathways and cellular events influenced by zonisamide-suppressed Apaf-1 and TNF-α in relation to AA. In the Apaf-1 protein–protein interaction (PPI) network, there were three shared proteins (GAPDH, TNF, IL1B) with the AD PPI network, whereas the TNF-α network included eight overlapping proteins (IL6, TLR4, INS, TNF, IL10, CD4, CRP, IL1B). TNF and IL-1B are proteins commonly obtained in both APAF-1- and TNF-α-associated interaction networks of AA. From a general perspective, both genes are commonly annotated with cytokine mechanisms, including cytokine activity, cytokine receptor binding, cytokine−cytokine receptor interaction, and molecular signaling activities, such as receptor−ligand activity signaling receptor activator/regulator activity. In the KEGGs analysis, APAF-1 revealed a remarkably significant annotation regarding antifolate resistance for AA. In the GO analysis results, APAF-1 was particularly associated with aspartic-type endopeptidase inhibitor activity and IL-1 receptor binding. On the other hand, concerning the relationship between TNF-α and AA, there were notably significant enrichments observed, specifically in choline binding and infections, including pathways related to Malaria and African trypanosomiasis (Figure 5).

## 4. Discussion

Heavy alcohol consumption poses a significant risk to human health and contributes to numerous diseases such as cancer, diabetes, neurological disorders, and cardiac problems [34]. The exact reasons for alcohol addiction (AA) are not fully understood, but various factors, including genetic predisposition, personality traits, and social interactions, contribute to alcohol consumption [35]. While the primary concern of alcohol consumption is its impact on the cerebrum, the cerebellum is also susceptible to its effects. Alcoholic cerebellar ataxia is one of the most common manifestations of alcohol’s impact on the cerebellum [36]. To demonstrate the induction of motor defects by ethanol (EtOH), we conducted open field maze tests and observed a decrease in locomotor ability, resulting in rats covering less area. However, zonisamide treatment ameliorated these defects following EtOH induction.

Heavy alcohol consumption leads to cerebellar cellular degeneration and atrophy due to alcohol toxicity. Upon ingestion, alcohol is metabolized to acetaldehyde by alcohol dehydrogenase and cytochrome enzymes and eventually to acetic acid [37]. Throughout these steps, free radicals are generated, and cells are predisposed to oxidative stress, leading to cellular pathology [38]. Various studies have shown that alcohol exposure impairs mitochondrial function and elevates free radical production [39]. Additionally, alcohol consumption induces neuroinflammation, contributing to alcohol neurotoxicity.

There is still no exact treatment for AA. Zonisamide has been shown to have positive impacts on central nervous system disorders such as Parkinson’s disease, dementia, migraines, and also AA as one of the current medications due to its unique mechanism of action [40,41]. Knapp et al. [25] reported significant reductions in alcohol consumption in experimental animals following the administration of zonisamide without altering the daily weight of the rats. Sarid-Segal et al. [42] have reported that a single dose of zonisamide reduced alcohol drinking in humans.

Since there is no study on the role of zonisamide in induced AA, we investigated its effects on cerebellar tissue, one of the components of the central nervous system. Studies have shown that zonisamide also has neuroprotective effects on nervous tissues. Wang et al. [43] reported that zonisamide reduced histopathological damage and cell apoptosis and supported motor neuron recovery in rats with degenerative myelopathy via the Fas and FasL signaling pathways. Owen et al. [44] indicated that zonisamide treatment decreased damage to pyramidal neurons and to the neuropil in hippocampal tissues after ischemic injury. In a study of cerebral ischemia–reperfusion injury, zonisamide ameliorated brain damage, promoting neuronal survival irrespective of its anticonvulsant effect [45]. In our study, similar to previous researchers, zonisamide contributed to histological improvements in neuronal regeneration and neuropil formation in cerebellar tissue via its neuroprotective effects.

Apoptotic protease activating factor-1 (APAF-1) is a crucial molecule that initiates the intrinsic or mitochondrial pathway of apoptosis within the cell [46]. Many studies have shown that EtOH induces apoptotic pathways via the activation of APAF-1. Oliveira et al. [47] evaluated the relationship between EtOH and apoptosis in rat cerebellar tissues. The authors found that EtOH induced apoptosis in Purkinje and cerebellar granule cells via the upregulation of caspase-3, which is a downstream molecule of APAF-1 in the apoptotic pathway. Similarly, Wang et al. [48] found that prolonged alcohol treatment elevated the level of Reactive Oxygen Species (ROS) and activated caspase-3, inducing apoptosis. A study showed that the administration of EtOH prevented cell proliferation, increased DNA fragmentation, and upregulated proapoptotic proteins [49]. Our results revealed that APAF-1 expression in the cerebellar cortex and white matter decreased in the EtOH + zonisamide group compared to the EtOH group. The downregulation of APAF-1 expression may indicate a protective effect of zonisamide treatment against apoptosis, potentially through the modulation of apoptotic pathways or the mitigation of cellular oxidant levels induced by alcohol.

Alcohol consumption alters immune cells and initiates an inflammatory response [50]. TNF-α is a proinflammatory cytokine involved in the inflammatory response. A study found that alcohol consumption led to impaired intestinal permeability, promoting the invasion of bacteria for the induction of inflammation [51]. Gonzales et al. [52] investigated 459 serum samples from hospitalized patients due to alcohol consumption and discovered that alcoholics exhibited the highest levels of TNF-α. Nanji et al. [53] revealed an increase in TNF-α levels in alcohol-induced liver injury. The authors claimed that TNF-α acts as a significant regulator at the molecular level of inflammation in liver cells by alcohol metabolism. Blood samples of patients with alcohol intake showed that TNF-α production was higher than in non-alcoholic patients [54]. In our study, we observed an increase in serum TNF-α levels in blood samples and TNF-α expression in the cerebellar tissue of the EtOH group. After zonisamide treatment, the EtOH + zonisamide group showed a decrease in both serum TNF-α levels and tissue TNF-α expression, particularly in the ganglionic and granular layers and within the neuroglia in the white matter. Our results suggest that alcohol consumption caused inflammation via the serum increase in TNF-α levels and also in tissue level. However, zonisamide restored the cerebellar tissue through its neuroprotective effects by suppressing the inflammation pathway.

The functional enrichment analysis was conducted to unravel the mechanisms by which zonisamide affects alcohol addiction, specifically in relation to APAF-1 and TNF-α. We observed a common and widespread impact on cytokine mechanisms and cellular signaling functions for both genes. Previous experimental studies have also demonstrated the effects of cytokines and intricate signaling pathways on AA [55,56,57]. For instance, studies have supported the involvement of the APAF-1 annotated Toll-like signaling pathway in neurotransmitter alteration, anxiety, and cognitive dysfunction in individuals with AA [56]. On the other hand, the relationship between antifolate resistance, which demonstrates a dominant association in APAF-1-associated pathways, and AA has not been well established. However, it is known that chronic alcohol exposure disrupts folate absorption and affects its circulation and hepatic uptake. Therefore, there may be a complex interaction between the folate mechanism, which plays a role in various biological processes such as methionine metabolism and epigenetic regulation [58], and APAF-1 and zonisamide. Studies on pathways associated with endopeptidases and IL1, which were found to be annotated with APAF-1, revealed that endopeptidases are effective in the tendency for alcohol consumption [59], and IL1 acts as a central mediator in the brain’s response to alcohol [57]. In addition, AA has the potential to harm individuals, leading to negative impacts on the gastrointestinal tract and the immune system. Such consequences might increase the vulnerability of patients to parasitic infections and influence the parasitic load [60,61]. TNFs play a dominant role in the response to parasitic and bacterial infections. Acetylcholine may modulate TNF-α release, and it has been demonstrated that an increase in TNF-α due to alcohol consumption leads to brain damage through glutamatergic excitotoxicity and demyelination in neurons [62,63,64]. All these findings support the connection between infections, choline binding, TNF-α, and AA, as identified in our enrichment analysis. Thus, zonisamide may exert its effects on AA through potential mechanisms involving APAF-1 and TNF-α. Collectively, these provide valuable insights into the potential molecular mechanisms underlying the effects of zonisamide on AA. Zonisamide’s suppression of APAF-1 and TNF-α, along with their associated pathways and functional annotations, suggests a multifaceted impact on immune regulation and cellular signaling in AA. The observed enrichments in pathways related to cytokines, immune responses, and infection pathways indicate a broader influence of zonisamide beyond traditional anti-alcohol effects, potentially involving the modulation of inflammatory processes and immune-mediated pathways.

## 5. Conclusions

Our findings conclude the impact of zonisamide on APAF-1 and TNF-α pathways in AA. Both genes are closely associated with cytokine mechanisms, highlighting their role in inflammation. APAF-1 is linked to antifolate resistance and interleukin-1 receptor binding, while TNF-α is implicated in choline binding and infections like malaria and African trypanosomiasis. Histochemical and immunostaining of cerebellar tissue sections revealed the protective effects of zonisamide against alcohol-induced neurodegeneration and neuroinflammation, as evidenced by reduced APAF-1 expression and inflammation marker TNF-α. Zonisamide treatment improved tissue integrity and myelination, suggesting its potential therapeutic utility in alcohol-dependence-associated neuropathology.

## Figures and Tables

**Table 1:** Total distance covered by rats in open field maze.

Groups	Distance (cm)	Multiple Comparisons
Sham	1301 (1161–1443)	*p* = 0.0006
EtOH	1017 (934–1100)	
EtOH + zonisamide	1174 (1135–1234)	

EtOH: ethanol, multiple comparisons were performed with Kruskal−Wallis, followed by Dunn’s test for binary comparison. Data are shown as median (IQR).

## Data Availability

All generated data were presented in the current study.
